# Ethical Considerations for Researchers’ Well-Being in the Development Process of Virtual Reality Scenarios for Child Emotional Maltreatment

**DOI:** 10.1007/s11948-026-00581-4

**Published:** 2026-02-09

**Authors:** Pia Keiski, Jari Kangas, Mikko Partanen, Tomi Nukarinen, Roope Raisamo, Eija Paavilainen

**Affiliations:** 1 Health Sciences Unit, Faculty of Social Sciences, Tampere University, Kalevantie 4, Tampere, 33014 Finland; 2 Faculty of Social Services and Health Care, Tampere University of Applied Sciences, Tampere, Finland; 3 Faculty of Information Technology and Communication Sciences, Tampere University, Kalevantie 4, Tampere, 33014 Finland; 4 Faculty of Social Sciences, Tampere University, Kalevantie 4, Tampere, 33014 Finland; 5 Etelä-Pohjanmaa Welfare Services County, Seinäjoki, Finland

**Keywords:** Ethics, Well-being, Virtual reality, Child, Parent, Emotional maltreatment

## Abstract

This paper aims to reflect on ethical considerations for the well-being of researchers involved in the development of virtual reality (VR) scenarios for parents who emotionally maltreat their children. Ethical considerations, particularly potential emotional strain, arose throughout the VR scenario development, including topic selection and content creation. However, despite the significance of these issues, existing literature addressing ethical challenges in researching sensitive topics from the researcher’s perspective has focused solely on data collection or analysis, overlooking the well-being of researchers involved in the development phase. To uphold high ethical standards, research should recognize and safeguard the well-being and safety of researchers throughout the study. In this methodological paper, the focus is on how an interdisciplinary team of researchers from technical and human sciences addressed the above concerns in biweekly reflective discussions. In these meetings, the team members shared their experiences and emotional reactions related to the development of VR scenarios simulating parents who were emotionally abusive toward their child. These reflective discussions created a secure environment for the researchers to process complex ethical issues and find emotional support, which is crucial for protecting their mental well-being from the outset of the project. The discussions contributed to strengthening the team’s overall well-being. In conclusion, this paper highlights the necessity of regular reflective meetings during the development phase to enhance the ethical quality of data gathering and reporting. In addition, engaging in team-based reflection improves researcher well-being and fosters ethical awareness. Reflective discussions have the potential to prevent vicarious traumatization by strengthening resilience when exposed to sensitive topics and addressing complex societal challenges.

## Introduction

Multidisciplinary research is increasing, particularly in healthcare. For instance, researchers from medicine, health and well-being sciences often collaborate with fellow researchers from information and communication technology (ICT) to develop new applications. However, health and medical researchers deal with more sensitive research topics and are used to dealing with emotionally distressing issues than ICT researchers, requiring special ethical considerations, particularly in the development phase. In addition, owing to their professional background, health and well-being researchers are more familiar with reflective discussions. During such collaborations, ICT researchers are also at risk of being exposed to distressing ethical situations, even if they themselves are not involved in actual sensitive data collection.

According to Baker et al. (2023), researcher well-being should be the responsibility of institutions, not just the individual researcher. It is crucial for researchers in multidisciplinary work to be able to and have time to reflect on their values and how they relate to those of others across disciplines (Hess et al., 2024). This can help create sustainable multidisciplinary research networks where the emphasis is on mutual support (Paavilainen et al., 2014).

Our research team includes members who are experts in technical or human sciences. Some of the researchers has experience in investigating child maltreatment and its prevention, and the others has experience in developing virtual reality (VR) technology. LaValle (2023) defines VR as “inducing targeted behavior in an organism by using artificial sensory stimulation, while the organism has little or no awareness of the interference.” This combination of expertise is necessary to determine the possibility of designing and implementing a VR tool for breaking the intergenerational cycle of child emotional maltreatment (CEM). The goal of the empirical study is to help emotionally abusive parents develop greater empathy and mentalization toward their child’s emotional experiences.

In empirical phase parents first view scenarios, and then they engage in guided discussions to process their experience, and mentalization is crucial part for this process. Mentalization, which is the ability to understand and reflect on others’ emotions, has been shown to reduce the risk of maltreatment (Fonagy, 2003; Gervinskaitė-Paulaitienė et al., 2023; Rosso, 2022). CEM is often passed down across generations through a cycle of parental learning, such as a lack of empathy (Buisman et al., 2024; Kaferly et al., 2020; McKenzie et al., 2021). It reflects the parents’ inability to meet their child’s emotional or developmental needs (Varma, 2015). It is noteworthy that parents often have histories of trauma and have experienced maltreatment themselves (Slep et al., 2022).

Ethical approval for the entire study was obtained from the relevant committee and participating research sites. This methodological paper aims to reflect on ethical considerations for the well-being of researchers involved in the development of VR scenarios for parents who emotionally maltreat their children.

## Background

The family and home environment should ideally serve as the primary social environment where children feel safe and protected by their parents. However, a Finnish national survey by Leppäkoski et al. (2021), including parents of 4-year-old children, found that 44% of the children had experienced at least one form of emotional maltreatment by their parents. CEM, which should become a public health priority, includes behaviors such as intimidation, threatening, belittling, name-calling, coercion, or indifference towards a child (Brassard et al., 2020). In recent years, increasing attention has been paid to the fact that CEM is equally harmful to children’s health and well-being as other, more physical forms of maltreatment (Nelson et al., 2020). However, despite the significance, the precise meaning of CEM is unclear, how parents and professionals define it, or how it differs from “poor parenting” (Brassard et al., 2020; Wolfe & McIsaac, 2011). Most importantly, there is also limited knowledge regarding CEM prevention. In addition, as evidenced in Baker et al.’s (2023) study (*N* = 538), the understanding of CEM appears to differ among welfare professionals; only 4 of the 18 items describing CEM were identified by most respondents as definitely involving CEM. In this context, VR can serve as a powerful tool to address the abstract and wicked problem of CEM by offering immersive experiences and raising awareness of the issue simultaneously.

As described above, the developed VR CEM prevention tool aims to provide parents with an immersive experience of CEM from the child’s perspective. Precisely, it serves as an “empathy machine,” providing emotionally powerful experiences that highlight how a child perceives emotional maltreatment by a parent. Previous studies have applied VR in various intervention contexts, such as self-harm prevention, teaching coping skills to aggressive youths, and learning non-violent responses to intimate partner violence (Christofi, 2021; Clus et al., 2018; Dellazizzo et al., 2019; Fromberger et al., 2018; Gonzalez-Liencres et al., 2020; Tan et al., 2022; Ventura et al., 2021). However, ethical considerations during the VR scenario development process, particularly from the researchers’ perspective, have received limited attention (e.g., Seinfeld et al., 2023).

Understanding participant perspectives is an important starting point for ethical evaluation (Finnish National Board on Research Integrity TENK, 2019). This meant carefully considering and ensuring that the stimuli presented to the participants were not overly upsetting to them (Shannon, 2022). Another important consideration is extending ethical evaluation to researchers’ well-being (Paavilainen et al., 2014), particularly in situations where the research team includes members who are not familiar with sensitive research topics. There is existing knowledge from the perspective of ensuring researcher safety and well-being during research and data analysis (Woodby et al., 2011), how researchers in qualitative research may encounter traumatic experiences while conducting interviews (e.g., Bashir, 2019; Bloor et al., 2010), and how researchers in quantitative research may experience distress (Bluvstein et al., 2021). Researching vulnerable groups may lead to emotional instability in researchers, feelings of powerlessness, and difficulty disconnecting from subjects; simultaneously, these experiences can foster positive outcomes such as personal growth (Garrels et al., 2022).

While the ethical risks associated with the use of VR systems have been extensively discussed (e.g., Kaimara et al., 2022; Kenwright, 2018), studies investigating the ethical risks that VR system developers face during the development work have not been reported. Furthermore, the ethics of generic software development and VR application development have been discussed, but only from the point of view of causing harm to others (e.g., ACM, 2024; Ramirez & LaBarge, 2018). VR application development often requires repeated iterations where developers test the immersive VR systems repeatedly, potentially exposing them to ethically harmful content.

Kumar and Cavallaro’s (2018) conceptual framework for researcher self-care identifies four types of emotionally demanding research experiences that require self-care: research on sensitive topics, research similar to the personal trauma previously experienced, research involving traumatic life events during the research process, and research on previously non-sensitive topics that unexpectedly uncover trauma. Investigating child maltreatment could be distressing for some, as it can bring back past memories, particularly if the researcher is familiar with the topic (Sikes & Hall, 2020). Researchers investigating violence need to acknowledge and address their emotional experiences during the research process; these reactions should be brought to the forefront and shared openly to maintain their ability to work with a sensitive subject (Markowitz, 2019). Even if a researcher is not in direct contact with the participants, a simple act of analysing sensitive data could still expose them to distress (Woodby et al., 2011). In our experience, this applies to the development phase itself.

Clinical supervision is not as commonly used in academia as it is in health and social care services. The purpose of clinical supervision is to support the professional development of employees, foster their well-being at work, and improve work practices (Markowitz, 2019). Reflective discussion, a core component of clinical supervision, allows participants to safely reflect on topics or issues they find emotionally burdening at work by helping them unpack the emotions they experienced in the actual situation and ensuring that they do not dwell on them or deal with them alone (Butterworth et al., 1999; Masamha et al., 2022). If distressing experiences are not unpacked and dealt with effectively, they can lead to vicarious traumatization. This means that a person’s inner experience can change as a result of deep empathic engagement with traumatized individuals, which may manifest as physiological arousal, emotional distress, negative changes in self-perception, or a desire to leave the profession (Saakvitne, 2002). In these discussions, researchers critically examine their own assumptions, beliefs, and judgments to explore how they affect them as well as their influence on the research process. Reflective discussions should be made mandatory in sensitive research projects to ensure researcher well-being and prevent vicarious traumatization (Molnar et al., 2017). Therefore, we employed reflective discussions as a method to mitigate the potential ethical risks of working with sensitive research topics on the researchers themselves.

## Reflective Discussions during the Development of VR Scenarios for CEM Studies

For the empirical study, we developed four VR scenarios that replicate some of the common CEM situations (Glaser, 2011; Wolfe & McIsaac, 2011): a verbally abusive conversation between the parents, a parent making the child feel guilty, a parent belittling and overestimating the child’s skills, and parents leaving the young child home alone (Table 1). The idea was to help parents view and experience the CEM situations from a child’s perspective, optimistically hoping that they reflect on their experience and eventually change their behavior. VR technology has been shown to create a credible, immersive experience from a child’s perspective (Banakou et al., 2013; Hamilton-Giachritsis et al., 2018).

**Table 1:** Four VR scenarios depicting some common CEM situations

Scenario	Description	Length
Scenario 1	A child wakes up to the sound of parents arguing in the next room and is forced to listen to offensive language and an escalating conflict. The child does not see the parents but hears them behind the wall.	1 min 51 s
Scenario 2	A child is eating at the table when a parent starts yelling at the child for being clumsy and makes the child feel guilty for something he/she is not responsible for.	1 min 20 s
Scenario 3	A parent and a child are in the living room (parents are divorced and living separately). The child is supposed to go to the other parent’s home for the weekend. The parent, who is concerned about the child’s safety and is worried about what could happen, talks about past family troubles and starts blaming the other parent for all of it. Irrespective of whether it is a real concern or simply a manipulation, the threat is slightly suggestive.	2 min 17 s
Scenario 4	A young child is left home alone while the parents go out in the evening, making the child feel unsafe. Before leaving, one of the parents belittles the child while talking on the phone with a friend. There is a slight hint of the parent blaming the child for not being what he/she wants them to be and for not yet knowing how to carry out tasks. As the sun sets, the child is sitting alone in the room, hearing the blaring sirens of emergency vehicles at a distance.	3 min

VR implementation is a standard work task for experienced software developers. Developers use standard software development tools to build any kind of VR environment, which were also used in our development work. First, the scenarios and interactions within the virtual environment were clearly specified in sufficient detail. Then, after gaining some experience with the implementation of the early versions, the specifications of the VR application were continuously refined and improved. To enable controlled collection of research material from the participants, we created four VR scenarios as part of the research design. All the scenarios were planned as short, scripted VR plays, each with a specific theme and a brief duration of only a few minutes. The themes of the VR scenarios were decided during reflective discussions with substance experts. Human researchers provided existing research material (Glaser, 2011; Wolfe & McIsaac, 2011) on common CEM situations to work through and discuss. The suitability of each theme for the implementations was discussed, including with professionals who have encountered parents who maltreat their children, and ultimately, the most promising themes were selected.

As in any VR application development, our goal during the software development process was to create VR scenarios that were as realistic as possible—that is, scenarios representing real maltreatment cases, so we had to make compromises on theme selection. The implementations had to be believable enough so that the participants would pay attention to the CEM case and not the VR, and this requirement affected the selections made. In addition, creating realistic avatar actions is challenging; therefore, we attempted to minimize their use and supplemented them with (mostly) other methods, primarily emotional speech, to convey the situation. All the VR experiences took place in a home environment, which all the participants could easily relate to. We employed voice actors to narrate stories for various scenarios.

Next, it was important that all the researchers understood emotional maltreatment from the point of view of a child to better determine which maltreating behavior would work in a VR scenario. This meant discussing various kinds of maltreatment cases and reflecting on them to find grounds for given scenarios while making use of the expertise of the technology researchers regarding what was possible to create in the VR environment. The discussions naturally exposed all the participants to descriptions of different CEM cases.

We expect the VR sessions to have fairly strong physiological effects on the participants; to confirm this, we prepared some biophysical measurements (e.g., heart rate variability) to obtain objective data. The biophysical data will not be used to determine the effectiveness of the study; rather, it was collected to gather more information on potential reactions. To ensure the participants’ and their families’ well-being, a researcher in human sciences was scheduled to contact the participants a week after the actual data collection to ask about the current situation in the family. In the meantime, the participants continued their therapeutic process.

The research team’s reflective discussions were held fortnightly as part of the iterative development process. They were initially part of internal project operations, but their role in promoting researchers’ well-being became evident already in the early stages of the development process. During these discussions, the scenarios were rewatched and discussed, and the related reactions were elaborated. The reflective discussions focused on the emotional and psychological impact of developing and repeatedly viewing the VR scenarios before the acceptance phase. We shared a wide range of emotional responses, with reactions varying between individuals and even within the same individual over time. A recurring theme in the discussions was the moral tension between the project’s goal of reducing CEM and the potential for inducing guilt in parents. The experience of possibly causing guilt and other distressing emotions was openly explored as part of the ethical reflection. From an ethical standpoint, this raises concerns about causing mental harm that exceeds the boundaries of everyday emotional experiences. Despite these challenges, the discussions consistently returned to the potential positive impact of the research - namely, improving children’s safety and well-being by helping parents gain insight into a child’s perspective.

The reflective discussions were intentionally unstructured, allowing all of us to freely share our thoughts and ask questions. All members of our research team participated as equals, and a consensus on each topic was built collaboratively. At the end of each discussion, we ensured that no one experienced excessive discomfort. Furthermore, during the discussions, the team reviewed the severity of CEM cases, and based on that, considered what kind of scenario would be feasible to implement. The maltreatment behavior had to be significant enough yet still tolerable for parents. A thorough ethical discussion was necessary to ensure that the benefits of the achieved outcomes outweigh the possible harm caused to the empirical study participants. In reflective discussions, researchers carefully reflected on what kinds of scenarios would be tolerable for the parents. This evaluation was continuously carried out alongside the development process by estimating the appropriate level of distress. The experience of human researchers was utilized in this context. Listening to and watching the VR scenarios also enabled preparing for the actual empirical part of study situation with the parents and ensured that ethical aspects were recognized and reflected as broadly as possible.

We found that listening to the voices was more distressing than watching the scenarios. We did not include any written descriptions in the VR experience because the aim was to create a scenario where the viewer understands the situation based on visual features alone and by listening to vocal content and other sounds provided, such as doors closing and dishes falling. We created the atmosphere of the VR scenarios and an understanding of what was happening through the vocal content of virtual characters rather than the characters’ movements, gestures, and facial expressions. While visible avatars were used in the final VR implementations, they were deliberately made to serve as somewhat passively moving actors in the scenarios.

When compiling the speech material, the general theme and tone of the speech samples were carefully controlled (what the situation entails, what to talk about, and how to talk about it); however, the voice actors had significant creative control over how they interpreted and performed their lines. The actors first created some test samples, which were reviewed by the development team for feedback before creating the final output. The vocal material included, for instance, a short sample of the parents fiercely arguing with each other, and another of a parent shouting at the child for being clumsy. The content of the voice samples was quite intense, which could be considered traumatizing. Asking the actors to provide vocal content naturally exposes them to the intense experiences depicted by the CEM cases when they had to rehearse and deliver their performances. However, the actors used techniques they had learnt during their education to unpack the burdening experiences related to delivering the voice recordings.

Once the VR implementations were finalized (including combining the visuals with the voice recordings and preparing measurements) and before starting the real data collection in experiments with real-life participants, we piloted the completed VR scenarios with a few professional therapists who had prior experience with CEM prevention. Their reactions to the VR scenarios were important indicators of the suitability and effectiveness of the solution. We were prepared to make final corrections to the scenarios based on their comments. In the process, the therapists were also exposed to the selected CEM experiences.

## Ethical Considerations Concerning the Researchers’ Well-Being

In research involving emotionally sensitive topics, such as VR development for CEM, ethical considerations must extend beyond protecting the participants to include the well-being of the researchers themselves. Researcher well-being is a critical ethical concern because it directly affects the integrity, safety, and sustainability of the research process. A reflective attitude, in turn, enhances a researcher’s ethical behavior by improving their perception of the research process and potential ethical issues, leading to high-quality research and a better understanding of the study’s multifaceted aspects (Karcher et al., 2024; Probst & Bernson, 2013). Likewise, we focused on the emotional well-being of the interdisciplinary research team during the VR development phase.

First, owing to the immersive nature of VR, which positions the viewer in the child’s perspective, it evoked strong emotional reactions even among the team members. Second, all researchers have personal histories, and many could relate to the emotional discomfort portrayed in the scenarios. Consequently, this identification with the child’s experience made the development process even more ethically sensitive. Therefore, to mitigate these risks, we implemented regular reflective discussions as a core method. These unstructured meetings created a safe and supportive environment for team members to share their thoughts, reactions, and vulnerabilities. The discussions resembled clinical supervision and promoted peer-to-peer support, emotional connectedness, and mutual understanding (AbiNader et al., 2023; Schulz et al., 2022). The process helped build trust and resilience within the team, which is essential before entering the data collection phase.

Furthermore, the emotional impact of the scenarios highlighted the potential for vicarious traumatization, a known risk in sensitive research (Molnar et al., 2017). By openly discussing distressing experiences, the team fostered vicarious resilience—a process where researchers experience strength and personal growth by observing the ability of others to cope with adversity (Engstrom et al., 2008). Listening to audio materials from the scenarios also served as a preparatory and reflective practice, enhancing emotional readiness and ethical awareness (Grill et al., 2014).

Reflection was not only a coping mechanism but also a learning process. It deepened the team’s understanding of the phenomenon and helped anticipate participants’ emotional responses (Kumagai & Naidu, 2015). This insight is ethically valuable, as it allows researchers to evaluate the justification of the study and balance potential distress against the intended benefits, such as reducing CEM.

## Conclusions

Researcher safety and well-being should be a crucial aspect of ethical review processes, **particularly for studies involving sensitive**,** distressing topics**. This critical, yet often overlooked, ethical responsibility is essential for maintaining ethical standards in sensitive research. **Principal investigators must be prepared to implement support for the research team’s safety** and well-being throughout the project. To achieve this, regular meetings, including reflective discussions, should be held. These sessions should allow team members to engage with potentially distressing materials and share their reactions.

**Building trust** is essential to foster open, confidential, and emotionally supportive discussions. This approach helps ensure that **high ethical standards** are upheld throughout all phases of sensitive research. Reflective discussions not only **protect researchers but also enhance the ethical quality** and depth of the research itself. They can also support researchers’ continued willingness to engage with emotionally demanding topics in the future.

## Data Availability

There is no data included to this manuscript.

## References

[CR1] AbiNader, M. A., Messing, J. T., Pizarro, J., Kappas Mazzio, A., Turner, B. G., & Tomlinson, L. (2023). Attending to our own trauma: Promoting vicarious resilience and preventing vicarious traumatization among researchers. *Social Work Research*, *47*(4), 237–249. 10.1093/swr/svad016

[CR2] ACM (Association for Computing Machinery) (2024). *ACM code of ethics and professional conduct*. https://www.acm.org/code-of-ethics

[CR3] Baker, S., Bellemore, P., & Morgan, S. (2023). Researching in fragile contexts: Exploring and responding to layered responsibility for researcher care. *Women’s Studies International Forum*, *98*, 102700. 10.1016/j.wsif.2023.102700

[CR4] Banakou, D., Groten, R., & Slater, M. (2013). Illusory ownership of a virtual child body causes overestimation of object sizes and implicit attitude changes. *Proceedings of the National Academy of Sciences of the United States of America*, *110*(31), 12846–12851. 10.1073/pnas.1306779110 23858436 10.1073/pnas.1306779110 PMC3732927

[CR5] Bashir, N. (2019). The qualitative researcher: The flip side of the research encounter with vulnerable people. *Qualitative Research*, *20*(5), 667–683. 10.1177/1468794119884805

[CR7] Bloor, M., Fincham, B., & Sampson, H. (2010). Unprepared for the worst: Risks of harm for qualitative researchers. *Methodological Innovations*, *5*(1), 45–55. 10.4256/mio.2010.0009

[CR8] Bluvstein, I., Ifrah, K., Lifshitz, R., Markovitz, N., & Shmotkin, D. (2021). Vulnerability and resilience in sensitive research: The case of the quantitative researcher. *Journal of Empirical Research on Human Research Ethics*, *16*(4), 396–402. 10.1177/15562646211027418 34180723 10.1177/15562646211027418

[CR9] Brassard, M. R., Hart, S. N., & Glaser, D. (2020). Psychological maltreatment: An international challenge to children’s safety and well being. *Child Abuse & Neglect*, *110*(Pt 1), 104611. 10.1016/j.chiabu.2020.104611 32660756 10.1016/j.chiabu.2020.104611

[CR10] Buisman, R. S. M., Compier-de Block, L. H. C. G., Bakermans-Kranenburg, M. J., Pittner, K., van den Berg, L. J. M., Tollenaar, M. S., Elzinga, B. M., Voorthuis, A., Linting, M., & Alink, L. R. A. (2024). The role of emotion recognition in the intergenerational transmission of child maltreatment: A multigenerational family study. *Child Abuse & Neglect*, *149*, 106699. 10.1016/j.chiabu.2024.106699 38417291 10.1016/j.chiabu.2024.106699

[CR11] Butterworth, T., Carson, J., Jeacock, J., White, E., & Clements, A. (1999). Stress, coping, burnout and job satisfaction in British nurses: Findings from the clinical supervision evaluation project. *Stress Medicine*, *15*(1), 27–33.

[CR13] Christofi, M. (2021). *Virtual reality as a medium for attitude change,* Doctoral dissertation, Department of Multimedia and Graphic Arts, Faculty of Fine and Applied Arts, Cyprus University of Technology.

[CR12] Clus, D., Larsen, M. E., Lemey, C., & Berrouiguet, S. (2018). The use of virtual reality in patients with eating disorders: Systematic review. *Journal of Medical Internet Research*, *20*(4), e157. 10.2196/jmir.7898 29703715 10.2196/jmir.7898 PMC5948410

[CR14] Dellazizzo, L., Potvin, S., Bahig, S., & Dumais, A. (2019). Comprehensive review on virtual reality for the treatment of violence: Implications for youth with schizophrenia. *NPJ Schizophrenia*, *5*(1), 11. 10.1038/s41537-019-0079-7 31337763 10.1038/s41537-019-0079-7 PMC6650426

[CR15] Engstrom, D., Hernandez, P., & Gangsei, D. (2008). Vicarious resilience: A qualitative investigation into its description. *Traumatology*, *14*(3), 13–21. 10.1177/1534765608319323

[CR16] Finnish National Board on Research Integrity TENK (2019). The ethical principles of research with human participants and ethical review in the human sciences in Finland. Finnish National Board on Research Integrity TENK Guidelines 2019 (pdf), Publications of the Finnish National Board on Research Integrity TENK 3/2019, 2nd revised edition–Finland. https://tenk.fi/en/advice-and-materials/guidelines-ethical-review-human-sciences

[CR17] Fonagy, P. (2003). Towards a developmental understanding of violence. *British Journal of Psychiatry*, *183*(3), 190–192. 10.1192/bjp.183.3.190 10.1192/bjp.183.3.190 12948988

[CR18] Fromberger, P., Jordan, K., & Müller, J. (2018). Virtual reality applications for diagnosis, risk assessment and therapy of child abusers. *Behavioral Sciences & the Law*, *36*(2), 235–244. 10.1002/bsl.2332 29520819 10.1002/bsl.2332

[CR19] Garrels, V., Skåland, B., & Schmid, E. (2022). Blurring boundaries: Balancing between distance and proximity in qualitative research studies with vulnerable participants. *International Journal of Qualitative Methods*, *21*. 10.1177/16094069221095655

[CR20] Gervinskaitė-Paulaitienė, L., Byrne, G., & Barkauskienė, R. (2023). Mentalization-based parenting program for child maltreatment prevention: A pre–post study of 12-week Lighthouse Group Program. *Children*, *10*(6), 1047. 10.3390/children10061047 37371278 10.3390/children10061047 PMC10297588

[CR21] Glaser, D. (2011). How to deal with emotional abuse and neglect: Further development of a conceptual framework (FRAMEA). *Child Abuse & Neglect*, *35*(10), 866–875. 10.1016/j.chiabu.2011.08.002 22014553 10.1016/j.chiabu.2011.08.002

[CR22] Gonzalez-Liencres, C., Zapata, L. E., Iruretagoyena, G., Seinfeld, S., Perez-Mendez, L., Arroyo-Palacios, J., Borland, D., Slater, M., & Sanchez-Vives, M. V. (2020). Being the victim of intimate partner violence in VR. *Frontiers in Psychology*, *11*, 820. 10.3389/fpsyg.2020.00820 32457681 10.3389/fpsyg.2020.00820 PMC7225265

[CR23] Grill, C., Ahlborg Jr., G., & Wikström, E. (2014). Health care managers learning by listening to subordinates’ dialogue training. *Journal of Health Organization and Management*, *28*(3), 437–454. 10.1108/JHOM-01-2013-0010 25080654 10.1108/JHOM-01-2013-0010

[CR24] Hamilton-Giachritsis, C., Banakou, D., Garcia Quiroga, M., Giachritsis, C., & Slater, M. (2018). Reducing risk and improving maternal perspective-taking and empathy using virtual embodiment. *Scientific Reports*, *8*(1), 2975. 10.1038/s41598-018-21036-2 29445183 10.1038/s41598-018-21036-2 PMC5813089

[CR25] Hess, J. L., Sanders, E., Fore, G. A., Coleman, M., Price, M., Nyarko, S., & Sorge, B. (2024). Transforming ethics education through a faculty learning community: I’m coming around to seeing ethics as being maybe as important as calculus. *Science and Engineering Ethics*, *30*(5), 40. 10.1007/s11948-024-00503-2 39251460 10.1007/s11948-024-00503-2 PMC11384631

[CR26] Kaferly, J., Furniss, A., & Allison, M. A. (2020). Transmission of intergenerational parenting attitudes and natural mentorship: Associations within the LONGSCAN population. *Child Abuse & Neglect*, *108*, 104662. 10.1016/j.chiabu.2020.104662 32861028 10.1016/j.chiabu.2020.104662

[CR27] Kaimara, P., Oikonomou, A., & Deliyannis, I. (2022). Could virtual reality applications pose real risks to children and adolescents? A systematic review of ethical issues and concerns. *Virtual Reality*, *26*(2), 697–735. 10.1007/s10055-021-00563-w 34366688 10.1007/s10055-021-00563-w PMC8328811

[CR28] Karcher, K., Mccuaig, J., & King-Hill, S. (2024). (Self-) reflection / reflexivity in sensitive, qualitative research: A scoping review. *International Journal of Qualitative Methods*, *23*. 10.1177/16094069241261860

[CR55] Kenwright, B. (2018). Virtual reality: Ethical challenges and dangers. *IEEE**Technology and Society Magazine, 37*(4), 20–25. 10.1109/MTS.2018.2876104

[CR30] Kumagai, A. K., & Naidu, T. (2015). Reflection, dialogue, and the possibilities of space. *Academic Medicine*, *90*(3), 283–288. 10.1097/ACM.0000000000000582 25426737 10.1097/ACM.0000000000000582

[CR31] Kumar, S., & Cavallaro, L. (2018). Researcher self-care in emotionally demanding research: A proposed conceptual framework. *Qualitative Health Research*, *28*(4), 648–658. 10.1177/1049732317746377 29224510 10.1177/1049732317746377

[CR32] LaValle, S. M. (2023). *Virtual reality*. Cambridge University Press. http://www.lavalle.pl/vr/

[CR33] Leppäkoski, T., Vuorenmaa, M., & Paavilainen, E. (2021). Psychological and physical abuse towards four-year-old children as reported by their parents: A National Finnish survey. *Child Abuse & Neglect*, *118*, 105127. 10.1016/j.chiabu.2021.105127 34139384 10.1016/j.chiabu.2021.105127

[CR34] Markowitz, A. (2019). The better to break and bleed with: Research, violence, and trauma. *Geopolitics*, *26*(1), 94–117. 10.1080/14650045.2019.1612880

[CR35] Masamha, R., Alfred, L., Harris, R., Bassett, S., Burden, S., & Gilmore, A. (2022). Barriers to overcoming the barriers’: A scoping review exploring 30 years of clinical supervision literature. *Journal of Advanced Nursing*, *78*(9), 2678–2692. 10.1111/jan.15283 35578563 10.1111/jan.15283 PMC9546137

[CR36] McKenzie, E. F., Thompson, C. M., Hurren, E., Tzoumakis, S., & Stewart, A. (2021). Who maltreats? Distinct pathways of intergenerational (dis)continuity of child maltreatment. *Child Abuse & Neglect*, *118*, 105105. 10.1016/j.chiabu.2021.105105 34051487 10.1016/j.chiabu.2021.105105

[CR37] Molnar, B. E., Sprang, G., Killian, K. D., Gottfried, R., Emery, V., & Bride, B. E. (2017). Advancing science and practice for vicarious traumatization/secondary traumatic stress. A research agenda. *Traumatology*, *23*(2), 129–142. 10.1037/trm0000122

[CR38] Nelson, C. A., Scott, R. D., Bhutta, Z. A., Harris, N. B., Danese, A., & Samara, M. (2020). Adversity in childhood is linked to mental and physical health throughout life. *Bmj*, *371*, m3048–m3048. 10.1136/bmj.m3048 33115717 10.1136/bmj.m3048 PMC7592151

[CR39] Paavilainen, E., Lepistö, S., & Flinck, A. (2014). Ethical issues in family violence research in healthcare settings. *Nursing Ethics*, *21*(1), 43–52. 10.1177/0969733013486794 23793068 10.1177/0969733013486794

[CR40] Probst, B., & Berenson, L. (2013). The double arrow: How qualitative social work researchers use reflexivity. *Qualitative Social Work*, *13*(6), 813–827. 10.1177/1473325013506248

[CR41] Ramirez, E. J., & LaBarge, S. (2018). Real moral problems in the use of virtual reality. *Ethics and Information Technology*, *20*(4), 249–263. 10.1007/s10676-018-9473-5

[CR42] Rosso, A. M. (2022). When parents fail to mind the child: Lower mentalizing in parents who maltreat their children. *Frontiers in Psychology*, *13*, 853343. 10.3389/fpsyg.2022.853343 35903725 10.3389/fpsyg.2022.853343 PMC9317950

[CR43] Saakvitne, K. W. (2002). Shared trauma: The therapist’s increased vulnerability. *Psychoanalytic Dialogues*, *12*(3), 443–449. 10.1080/10481881209348678

[CR44] Schulz, P., Kreft, A. K., Touquet, H., & Martin, S. (2022). Self-care for gender-based violence researchers – Beyond bubble baths and chocolate pralines. *Qualitative Research*, *23*(5), 1461–1480. 10.1177/14687941221087868

[CR45] Seinfeld, S., Hortensius, R., Arryo-Palacios, J., Iruretagoyena, G., Zapata, L. E., de Gelder, B., Slater, M., & Sanchez-Vives, M. V. (2023). Domestic violence from a child perspective: Impact of an immersive virtual reality experience on men with a history of intimate partner violent behavior. *Journal of Interpersonal Violence*, *38*(3-4), 2654–2682. 10.1177/08862605221106130 35727942 10.1177/08862605221106130

[CR46] Shannon, E. R. (2022). Safeguarding and agency: Methodological tensions in conducting research with survivors of sexual violence in universities. *Social Sciences*, *11*(8), 350. 10.3390/socsci11080350

[CR47] Sikes, P., & Hall, M. (2020). Too close for comfort? Ethical considerations around safeguarding the emotional and mental wellbeing of researchers using auto/biographical approaches to investigate ‘sensitive’ topics. *International Journal of Research & Method in Education*, *43*(2), 163–172. 10.1080/1743727X.2019.1636025

[CR49] Slep, A. M. S., Glaser, D., & Manly, J. T. (2022). Psychological maltreatment: An operationalized definition and path toward application. *Child Abuse & Neglect*, *134*, 105882. 10.1016/j.chiabu.2022.105882 36137405 10.1016/j.chiabu.2022.105882

[CR50] Tan, M. C. C., Chye, S. Y. L., & Teng, K. S. M. (2022). In the shoes of another: Immersive technology for social and emotional learning. *Education and Information Technologies*, *27*(6), 8165–8188. 10.1007/s10639-022-10938-4 35261549 10.1007/s10639-022-10938-4 PMC8890956

[CR51] Varma, V. (2015). The secret life of children who have experienced emotional abuse. In *The secret life of vulnerable children* (pp. 75–100). Taylor & Francis Group.

[CR52] Ventura, S., Cardenas, G., Miragall, M., Riva, G., & Baños, R. (2021). How does it feel to be a woman victim of sexual harassment? The effect of 360°-video-based virtual reality on empathy and related variables. *Cyberpsychology Behavior and Social Networking*, *24*(4), 258–266. 10.1089/cyber.2020.0209 33085513 10.1089/cyber.2020.0209

[CR53] Wolfe, D. A., & McIsaac, C. (2011). Distinguishing between poor/dysfunctional parenting and child emotional maltreatment. *Child Abuse & Neglect*, *35*(10), 802–813. 10.1016/j.chiabu.2010.12.009 22015202 10.1016/j.chiabu.2010.12.009

[CR54] Woodby, L. L., Williams, B. R., Wittich, A. R., & Burgio, K. L. (2011). Expanding the notion of researcher distress: The cumulative effects of coding. *Qualitative Health Research*, *21*(6), 830–838. 10.1177/1049732311402095 21393618 10.1177/1049732311402095

